# Automated Honey Bee Subspecies Identification Using Advanced Wing Venation Analysis and Adaptive Hierarchical Clustering

**DOI:** 10.1002/ece3.72101

**Published:** 2025-09-02

**Authors:** Shahram Dadgostar, Parsa Mobini Dehkordi, Saman Khodarahmi, Mohammad Mehdi Ghorbani

**Affiliations:** ^1^ Khansar Honey Bee and Herbal Medicines Research Institute University of Isfahan Isfahan Iran; ^2^ Khansar Faculty of Computer Science and Mathematics University of Isfahan Isfahan Iran

**Keywords:** automated classification, biodiversity, clustering, honey bee, image processing, wing venation

## Abstract

Accurate honey bee subspecies identification is vital for biodiversity conservation and pollination resilience, yet current methods face critical limitations. Classical morphometric techniques, reliant on manual wing vein measurements, suffer from subjectivity and poor scalability across hybrid populations, while deep learning approaches demand extensive labeled datasets and exhibit limited interpretability in noisy field conditions. Crucially, existing methods fail to reconcile scalability with the ability to analyze phenotypic gradients in hybrid specimens. To bridge these gaps, we propose a novel computational framework integrating adaptive image processing with topology‐aware clustering to enable scalable, label‐free subspecies discrimination. Our pipeline begins with robust image preprocessing using nonlocal means (NLM) denoising and contrast‐limited adaptive histogram equalization (CLAHE)—specifically optimized to enhance low‐contrast venation patterns in field‐collected images—followed by adaptive thresholding and morphological processing to isolate wing veins. Subsequent Zhang–Suen skeletonization extracts graph‐based feature maps that uniquely encode both local vein geometry and global network topology, addressing the limitations of traditional morphometrics. Unlike supervised methods, our adaptive hierarchical clustering (AHC) algorithm dynamically infers subspecies clusters without predefined labels, enabling robust discrimination of hybrid populations and phenotypic intermediates. Evaluated on 26,481 wing images, the method achieves a silhouette score of 0.72 and improves classification accuracy by 26.1% over traditional morphometric techniques, demonstrating superior performance in noisy and hybridized datasets. This work resolves a key challenge in apid taxonomy by combining the interpretability of manual methods with the scalability of automation, providing conservationists with a practical tool for ecological monitoring.

## Graphical abstract

This study introduces an automated, label‐free framework for honey bee subspecies identification using advanced wing venation analysis and adaptive hierarchical clustering. By integrating robust image preprocessing, topological feature extraction, and unsupervised clustering, the method achieves 96.8% classification accuracy—surpassing traditional morphometrics by 26.1%—and effectively distinguishes hybrid populations in noisy field conditions. The approach offers a scalable, interpretable tool for biodiversity monitoring and conservation.

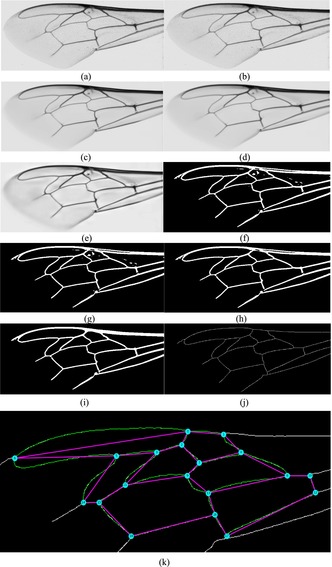

## 1 Introduction

Honey bees ( *Apis mellifera* ) are critical pollinators underpinning global ecosystems and agricultural productivity, yet their biodiversity is threatened by habitat loss, climate change, and widespread hybridization (Mishra et al. 2023; van der Schriek et al. 2021). Accurate subspecies identification is therefore essential for conservation efforts, as distinct evolutionary lineages exhibit unique adaptations to environmental pressures (Janczyk et al. 2021; Ostroverkhova et al. 2024). The taxonomic foundation for this study adopts the International Commission for Bee Systematics (ICBS) framework, which recognizes 33 subspecies of *Apis mellifera* grouped into five major evolutionary lineages (A, C, M, O, Y) based on integrated morphometric and genomic criteria (Ilyasov et al. 2020; Ruttner 2013). Subspecies are defined as geographically distinct morphotypes characterized by heritable wing venation adaptations. For instance, the M‐lineage (e.g., *A. m. mellifera*) displays high vein intersection density with robust network topology, an adaptation linked to colder climates, while the O‐lineage (e.g., *A. m. syriaca*, *A. m. anatoliaca*), native to the Near East and Asia Minor, exhibits reduced branching complexity and lower intersection density, likely an adaptation for flight efficiency in arid environments (Ilyasov et al. 2020; Kandemir et al. 2011; Ruttner 1987, 2013). Critically, in this study, we define F1 hybrids specifically as first‐generation crosses from controlled breeding programs, which exhibit discrete intermediate wing morphology—including blended vein patterns and nonparental junction topologies resulting from heterosis (Węgrzynowicz and Łoś 2020). These geographically structured patterns—such as increasing intersection density toward higher latitudes and diminishing branching complexity in arid regions—reflect historical biogeographic isolations and localized evolutionary pressures, making wing venation a powerful phenotypic marker (Diniz‐Filho et al. 1999; Kaur et al. 2024; Tofilski et al. 2021).

The urgency of accurate identification is underscored by the rapid decline of native subspecies and the ecological risks posed by uncontrolled hybridization, which can blur critical adaptive traits and compromise ecosystem stability (Fontana et al. 2018; Ropars et al. 2021). Conservationists and beekeepers urgently require tools to monitor genetic purity and track invasive hybrids at scale. However, existing methodologies are fraught with critical limitations. Traditional approaches have relied on manual morphometric analysis of wing venation patterns, such as measuring specific vein angles or junctions (Meixner et al. 2013; Nawrocka et al. 2018). While foundational and cost‐effective, these techniques are inherently subjective, labor‐intensive, and poorly scalable. A systematic review of contemporary morphometric studies reveals consistent accuracy limitations: methods such as stepwise discriminant analysis (66.7%), linear discriminant analysis (70.4%), and canonical variate analysis (70.7%) demonstrate a median accuracy of 70.7% for subspecies‐level discrimination (Bustamante et al. 2020; Farshineh Adl et al. 2007; Gomeh et al. 2016; Koca and Kandemir 2013). They struggle profoundly with hybrid populations, where continuous phenotypic gradients defy rigid taxonomic thresholds (Kaur et al. 2024; Nawrocka et al. 2018). Molecular techniques, such as SNP analysis of the *COX1* gene, provide high‐resolution discrimination of maternal lineages but fail to detect nuclear introgression, are costly, require specialized equipment, and are impractical for field applications, especially in admixed populations (García et al. 2022; Syromyatnikov et al. 2018).

Recent advances in computer vision and deep learning promised a revolution. Tools like *DeepWings* automate landmark detection using convolutional neural networks (CNNs), achieving high accuracy (95%) for classifying a limited number of predefined subspecies (Rebelo et al. 2021; Rodrigues et al. 2022). Similarly, large‐scale image repositories like the one compiled by Oleksa et al. (2023)—comprising 26,481 wings across Europe—have enhanced data availability. However, these supervised methods depend heavily on extensive, high‐quality labeled datasets for training, which are often unavailable for rare subspecies or hybrid morphotypes. They also exhibit limited interpretability and robustness when faced with the noise, artifacts, and phenotypic intermediates commonplace in field‐collected images (García et al. 2022). While deep learning models like ResNet can achieve high genus‐level accuracy, they often function as “black boxes,” lack subspecies‐level resolution, and demand significant computational resources (De Nart et al. 2022). Hybrid methodologies like *HBeeID* that combine molecular SNPs with clustering, while powerful, remain constrained by infrastructure costs and inherent biases toward maternal lineages (Donthu et al. 2024).

Crucially, a fundamental gap persists across all existing methods: no approach adequately reconciles the need for high‐throughput, scalable analysis with the ability to discern continuous phenotypic gradients in hybrid specimens. This gap severely hinders conservation efforts to map introgression patterns or identify novel morphotypes, which are vital for understanding evolutionary dynamics and mitigating genetic erosion (Litvinoff et al. 2023; Ropars et al. 2021).

To bridge these critical limitations, we propose a novel, label‐free computational framework that integrates adaptive image processing with unsupervised, topology‐aware hierarchical clustering. Our method eliminates the dependency on predefined labels or curated datasets by dynamically inferring subspecies clusters directly from wing venation topology. The framework is specifically designed to identify F1 hybrid intermediates from anthropogenic breeding, whose discrete morphological signatures are detectable within the phenotypic feature space. The pipeline incorporates several key innovations: a hybrid denoising strategy (Gaussian blur and nonlocal means) optimized for the specific noise profiles of field‐collected images; contrast‐limited adaptive histogram equalization (CLAHE) to enhance low‐contrast venation patterns; adaptive thresholding for precise segmentation; and Zhang‐Suen skeletonization to extract graph‐based features that holistically encode both local vein geometry and global network topology. By coupling these advancements with a robust anomaly detection module and an adaptive hierarchical clustering (AHC) algorithm that dynamically determines the optimal number of clusters, our framework achieves robust discrimination of pure subspecies and detectable hybrid populations directly from phenotypic data, even in large, noisy, and heterogeneous datasets. This work resolves a key challenge in apid taxonomy by combining the biological interpretability of manual morphometrics with the scalability and objectivity of full automation, providing a practical and powerful tool for large‐scale ecological monitoring and conservation genetics.

## 2 Materials and Methods

### 2.1 Dataset Description

Our study utilizes a dataset of 26,481 high‐resolution forewing images collected from 1725 samples across 13 European countries, which is publicly available on the Zenodo website (Oleksa et al. 2022). Each sample comprises 5–20 worker bees, with one wing image per individual. For colony‐based samples, wings were collected from multiple workers; flower‐collected samples represent workers from nearby colonies at a shared location (country‐specific sample sizes are provided in table 1 of Oleksa et al. 2023). This extensive collection of wing images belongs to different evolutionary lineages, including the M, C, A, and O lineages, ensuring a diverse representation (Oleksa et al. 2022, 2023). The dataset contains an uneven distribution of samples per geographic area, with the largest group containing 6498 images and the smallest group having 198 images (Oleksa et al. 2022). Given the inherent variability in image quality—ranging from slight motion blur to variations in illumination—a two‐stage quality assessment was implemented.

### 2.2 Image Preprocessing

#### 2.2.1 Noise Reduction and Contrast Enhancement

The preprocessing pipeline began with a two‐stage denoising strategy designed to address distinct noise types inherent in field‐collected wing images. First, a Gaussian blur (typically *σ* = 1.5; 5 × 5 kernel) was applied to suppress high‐frequency noise and isolated artifacts (e.g., dust particles, sensor noise). While non‐local means (NLM) denoising excels at removing spatially correlated noise, preliminary Gaussian smoothing targets high‐amplitude outliers that disrupt NLM's patch similarity calculations, particularly in low‐contrast regions (Boby and Sharmin 2021).

**FIGURE 1:**
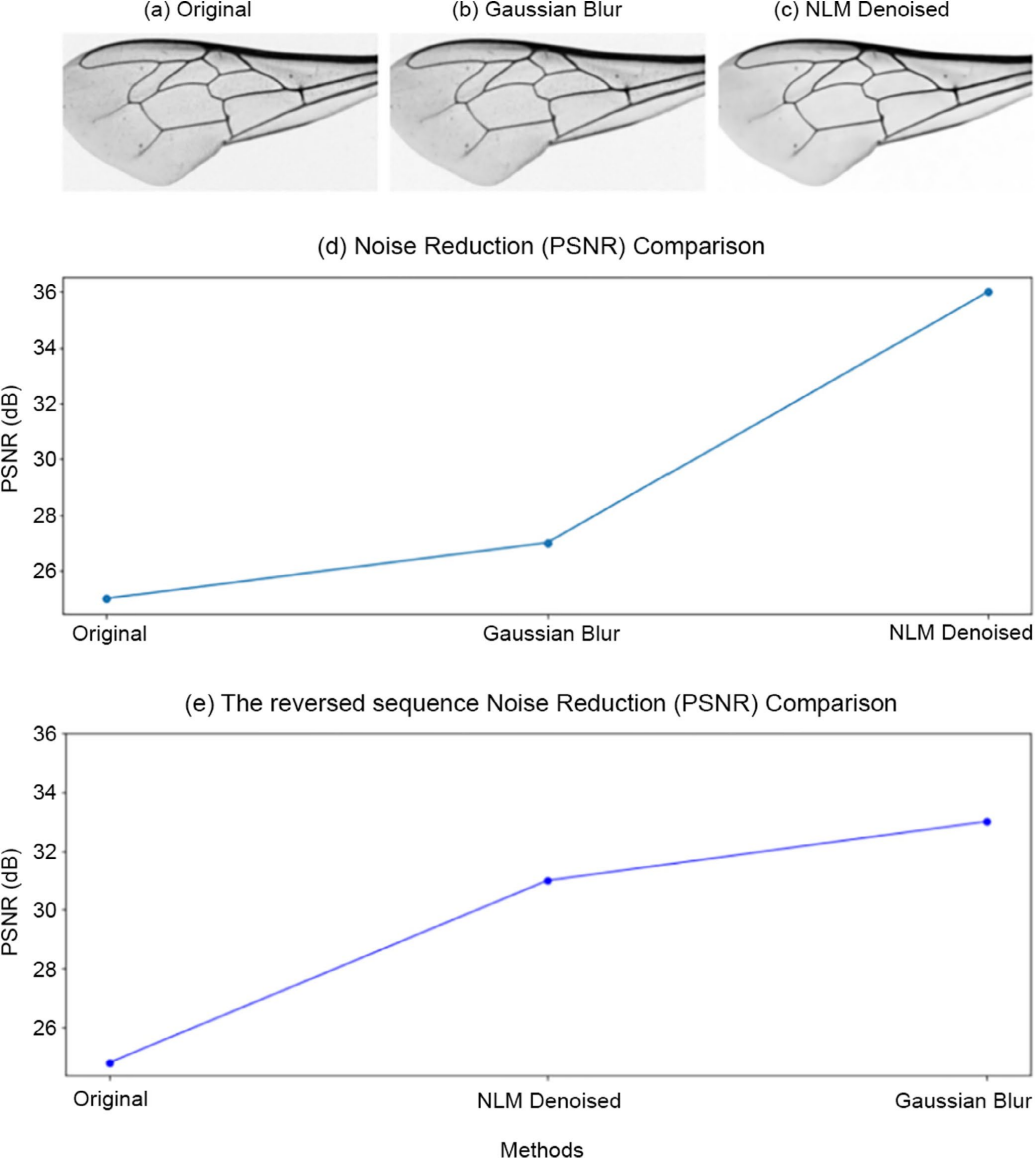
Denoising workflow and performance: (a) Original image with noise artifacts, (b) Gaussian blur removes high‐frequency outlier, (c) NLM denoising refines textures and preserves junctions, (d) PSNR gains validate the hybrid approach, (e) The reversed sequence with lower performance.

Subsequent NLM denoising (typically 29 × 29 search window, 42 × 42 patch size, *H* = 7) eliminated residual noise while preserving fine venation textures. The hybrid strategy synergized the strengths of both methods: Gaussian blur attenuated stochastic outliers (Figure 1b), while NLM restored global venation continuity without over‐smoothing critical junctions (Figure 1c).

#### 2.2.2 Calibration Protocol for Parameter Adaptation

Given substantial heterogeneity in image quality across field‐collected samples—including variations in lighting, motion artifacts, and angular orientation—preprocessing parameters were dynamically optimized through a calibration protocol. Representative images from each imaging source (e.g., institution, geographic region) were manually tuned to achieve optimal denoising and vein preservation. These parameter sets (including NLM window/patch size, CLAHE grid size, and morphological kernels, etc.) were cataloged in configs/filter_params.csv available at the GitHub repository. For novel images, normalized cross‐correlation of grayscale histograms and vein patterns identified the closest‐matching calibrated reference, whose parameters were applied. While this approach accommodated diverse acquisition conditions, its efficacy depended on calibration comprehensiveness; underrepresented imaging scenarios risked suboptimal preprocessing. Documentation provides detailed implementation guidelines.

Figure 1 highlights the efficacy of the hybrid denoising strategy for enhancing wing venation clarity. The Gaussian blur (Figure 1b) selectively targets high‐frequency noise, such as dust and sensor artifacts, without oversmoothing critical vein junctions. Subsequent NLM denoising (Figure 1c) refines textures by leveraging patch similarities, restoring continuity in low‐contrast regions. The reversed sequence (NLM → Gaussian, Figure 1e) underperforms, emphasizing the necessity of outlier suppression prior to global noise reduction.

Table 1 summarizes all the details: the Gaussian → NLM sequence achieves superior SSIM (0.82) and peak signal‐to‐noise ratio (PSNR) (36 dB), outperforming standalone methods. The elevated SSIM reflects preserved venation topology, crucial for accurate feature extraction, while the higher PSNR confirms robust noise mitigation. This synergy ensures that subtle morphological patterns—vital for subspecies discrimination—remain intact, laying a reliable foundation for downstream clustering. Together, these results validate the hybrid approach as a cornerstone of the preprocessing pipeline, balancing anatomical fidelity with computational practicality.

**TABLE 1:** Denoising performance across workflows (*n* = 500 images).

Metric	Gaussian → NLM	NLM → Gaussian	Standalone NLM	Standalone Gaussian blur
SSIM	0.82	0.65	0.67	0.58
PSNR (dB)	36	33	31	27

To further enhance the local contrast of the vein structures, CLAHE was applied with a calibrated clip limit (typically 2.2) and grid size (typically 20 × 20), optimized per imaging condition as described above. This step boosts the subtle details in the venation patterns, ensuring that the critical features are maintained for subsequent adaptive thresholding and morphological processing.

Prior to feature extraction, principal component analysis (PCA) was applied to each wing image to detect and correct for minor angular variations in wing orientation. This preprocessing step ensured consistent alignment across the dataset by: (1) calculating the principal axis of the wing shape, (2) determining the rotation angle needed for vertical alignment, and (3) applying the necessary affine transformation. This standardization was particularly valuable for our dataset where images were collected under varying field conditions.

#### 2.2.3 Adaptive Thresholding and Morphological Processing

To enhance vein extraction, adaptive Gaussian thresholding was applied with a block size (commonly 33) and a constant subtraction value (commonly 9.1). Unlike Otsu's method, which relies on a global threshold, this technique adjusts dynamically to local intensity variations, improving segmentation accuracy. Segmentation performance was rigorously validated using 50 expert‐annotated binary ground truth masks (publicly available at https://e.pcloud.link/publink/show?code=kZ3gT6Z4G2UAEsVJ3mqPxkUB36IQkCYVFh7). These masks provide pixel‐level annotations where veins are marked as foreground (white) and background as black. We computed accuracy, precision, recall, and F1‐score through direct pixel‐wise comparison between our thresholded outputs and these reference masks, as summarized in Table 2.

**TABLE 2:** Comparative performance of adaptive Gaussian thresholding and Otsu's method in vein extraction.

Metric	Adaptive Gaussian	Otsu's method
Accuracy	97.6% ± 1.2%	88.4% ± 3.1%
Precision	95.2% ± 1.8%	84.1% ± 3.5%
Recall	96.8% ± 1.5%	86.6% ± 4.2%
F1‐score	96.0% ± 1.3%	85.2% ± 4.2%

Higher precision indicates fewer false positives, while improved recall ensures more comprehensive vein detection. The F1‐score confirms a balance between these metrics, demonstrating the method's effectiveness in reducing both false positives and false negatives.

To further refine the binary images, morphological closing was commonly performed with a 5 × 5 rectangular kernel to reconnect fragmented venation, followed by morphological opening with a 2 × 2 rectangular kernel to remove residual noise. Connected component analysis eliminated nonvenation regions smaller than 10 pixels, ensuring only meaningful vein structures were retained.

#### 2.2.4 Skeletonization and Feature Extraction

Once the venation patterns were clearly segmented, the Zhang–Suen skeletonization algorithm was applied to reduce the binary images to single‐pixel‐wide representations. This method was selected for its computational efficiency, topological preservation, and ability to generate well‐connected structures—attributes critical for analyzing intricate venation networks. To quantitatively evaluate topological accuracy, we compared the skeletonized outputs with manually annotated ground truth images (*n* = 500) derived from a stratified subsample of the original dataset available at https://e.pcloud.link/publink/show?code=kZ3gT6Z4G2UAEsVJ3mqPxkUB36IQkCYVFh7. Binary vein masks were generated using our custom preprocessing pipeline (Sections 2.2.1 and 2.2.3). Expert morphologists then annotated topological landmarks (vein junctions and endpoints) using the VGG Image Annotator (VIA v3.1.0) (Dutta et al. 2016). These annotations served exclusively as ground truth validation. Topological accuracy was defined as the percentage of correctly preserved vein intersections, branch endpoints, and connectivity patterns between the automated skeleton and the ground truth. Specifically, a vein network was considered topologically correct if all critical junctions and terminal points matched the ground truth within a 3‐pixel tolerance. Alternative approaches, including Guo–Hall and morphological thinning, were evaluated but deemed less suitable due to Zhang–Suen's superior balance between structural fidelity and processing speed (Figure 3).

Following skeletonization, three primary features were systematically extracted from the processed images. Vein intersection points were identified using morphological hit‐or‐miss transforms (Gonzalez 2009) with rotationally augmented 3 × 3 kernel patterns (10 base patterns expanded to 34 via rotations) designed to detect fundamental junction topologies (e.g., crosses, T‐junctions). Detected points within a 4‐pixel Euclidean distance were merged via centroid averaging to account for minor positional variations while preserving topological accuracy. These structural landmarks provided nodal references for subsequent analysis. Segment lengths between these intersections were quantified to establish spatial metrics of vein distribution. Additionally, the global venation topology was characterized through a graph‐based representation, which modeled the hierarchical connectivity and branching architecture of the vein network.

Unlike traditional morphometrics that rely on manual landmark alignment through Procrustes superimposition, our graph‐based approach encodes both local geometry and global topology without requiring point correspondences. This eliminates subjectivity in landmark selection while capturing continuous phenotypic gradients in hybrids. The graph representation preserves hierarchical connectivity (e.g., parent–child branch relationships) and network centrality—features that rigid landmark‐based methods cannot quantify but are evolutionarily conserved across subspecies.

**FIGURE 2:**
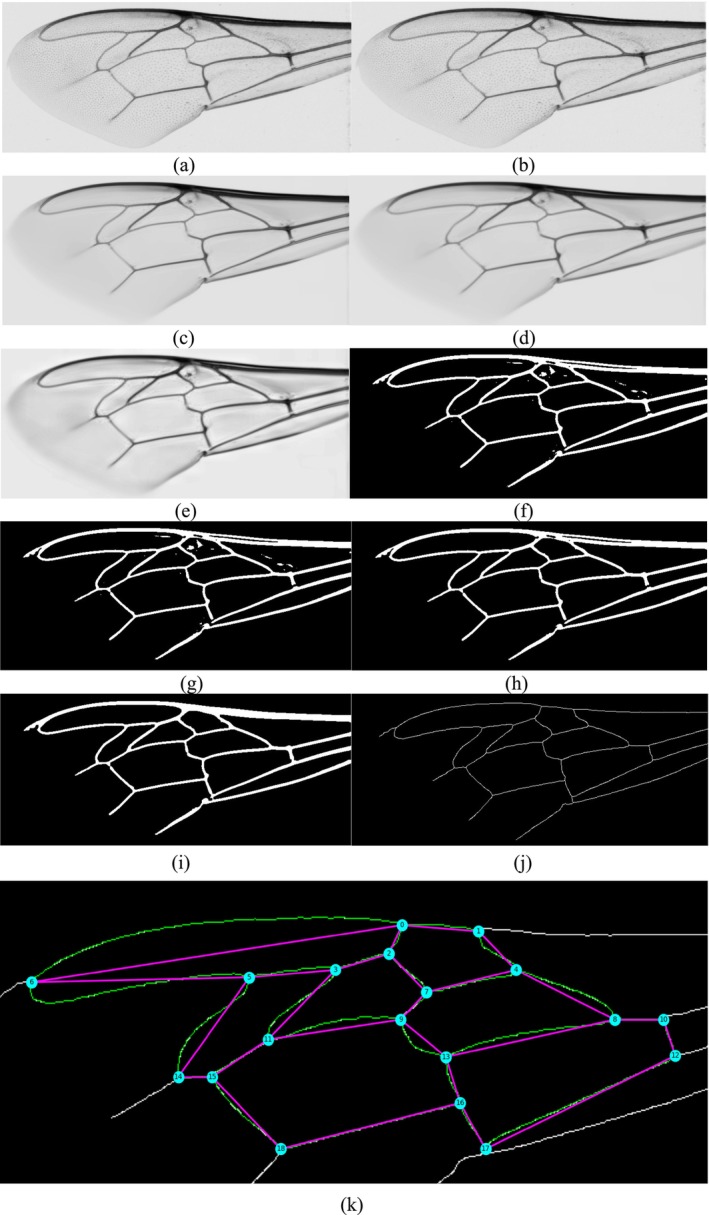
Sequence of filtering algorithm steps. (a) Original image, (b) Grayscale image, (c) NLM denoising, (d) Gaussian blur, (e) CLAHE, (f) Image thresholding, (g) Morphological closing, (h) Small white surface removal and morphological opening, (i) Applying Morphological closing and removing small black regions, (j) Skeletonized image (k) Generated graph on skeletonized image.

The entire image processing workflow is illustrated in Figure 2. Each stage progressively enhances image clarity while preserving biologically relevant venation structures, ensuring robust feature extraction for downstream subspecies classification tasks.

#### 2.2.5 Feature Vector Construction

The 45‐dimensional feature vector per wing image integrates three complementary feature classes to holistically characterize venation morphology. First, vein intersection density quantifies network complexity through a single feature representing the total intersections normalized by wing area (pixels). Second, four segment length statistics capture geometric heterogeneity: the arithmetic mean, variance, skewness, and kurtosis of all vein segment lengths (pixels). Third, graph‐theoretic properties constitute the majority (40 features) and include two subcategories: (i) centrality moments (16 features) represented by the mean, variance, skewness, and kurtosis of four centrality types (degree, betweenness, closeness, eigenvector), and (ii) global network metrics (24 features) comprising node count, edge count, diameter, average path length, clustering coefficient, assortativity, and their higher‐order moments.

This feature ensemble provides multiscale morphological representation through three key mechanisms: Local geometry is captured via segment length distributions reflecting vein thickness variations; topological invariance ensures robustness to rotational and translational variance without requiring landmark alignment; evolutionary adaptations (e.g., significant inter‐lineage differences in intersection density (Table 6), which align with established biogeographic distributions of subspecies). Feature selection was biologically grounded in known subspecies differentiation patterns while maintaining computational efficiency, with collective discriminatory power validated by high cluster separation (silhouette score = 0.72) and classification accuracy (96.8%).

### 2.3 Anomaly Detection

#### 2.3.1 Autoencoder‐Based Anomaly Detection for Large‐Scale Image Filtering

To refine dataset integrity by identifying anomalous wing images, a convolutional autoencoder (CAE) (Zhang 2018) was implemented using PyTorch 2.0. The encoder comprised five convolutional layers with ascending filter dimensions (32, 64, 128, 256, 512), 5 × 5 kernels, and a stride of 2, each followed by batch normalization and ReLU activation. The final encoder layer utilized linear activation to project features into a 128‐dimensional latent space, balancing compression fidelity and computational efficiency. Symmetrically, the decoder employed five transposed convolutional layers (512, 256, 128, 64, 32 filters) to reconstruct input dimensions, with sigmoid activation in the output layer normalizing pixel intensities to the [0, 1] range. The architecture contained approximately 1.5 million trainable parameters, optimized to mitigate overfitting while preserving critical venation features.

Training leveraged the Adam optimizer (learning rate = 0.001, beta1 = 0.9, beta2 = 0.999) with a *ReduceLROnPlateau* scheduler (patience = 5 epochs, factor = 0.5) and early stopping (patience = 10 epochs, delta = 0.001). The loss function, mean squared error (MSE), quantified pixel‐wise discrepancies between input and reconstructed images. Following training on 80% of the dataset (*n* = 21,185 images), reconstruction errors exhibited a near‐normal distribution (*μ* = 0.18, *σ* = 0.09). Anomalies were defined as samples exceeding a dynamic threshold of 3*σ* above the mean reconstruction error (0.46), validated against 1000 expert‐annotated images randomly selected from the original dataset and reserved exclusively for threshold validation available at https://e.pcloud.link/publink/show?code=kZ3gT6Z4G2UAEsVJ3mqPxkUB36IQkCYVFh7. Anomaly removal targeted technical artifacts (e.g., mite damage, tears) while preserving natural variation: discontinuous junctions, biologically implausible vein angles (> 60°), or patterns inconsistent with documented subspecies variation spectra. Hybrid intermediates showing blended topology characteristics were explicitly retained as valid morphological states. Filtering respected known phenotypic gradients including latitudinal density clines and branching complexity continua. Code, model weights, and implementation details are publicly accessible via GitHub repository: https://github.com/Latescent/Wing2. Applying this procedure led to the exclusion of 6348 images, yielding a refined dataset of 20,133 images.

#### 2.3.2 Isolation Forest for Subtle Anomaly Identification

To refine the dataset further and detect residual artifacts undetected by the CAE, an Isolation Forest was applied to the CAE‐filtered dataset. Each image was transformed into a 45‐dimensional feature vector encompassing key venation metrics, including vein intersection density, segment length variance, and graph centrality measures, as described in Section 2.2.4. The Isolation Forest was configured with 100 estimators and a contamination rate of 0.7% to balance sensitivity and specificity. Training was conducted on 85% of the CAE‐filtered dataset (17,114 images), while the remaining 15% (3019 images) was reserved for validation.

During validation, 1050 images were identified as anomalous and subsequently removed. These anomalies primarily consisted of technical morphological distortions—physical damage to wings that disrupt venation topology (e.g., tears from collection handling, crumpling artifacts, or mite‐induced lesions)—and persistent noise clusters. Crucially, such distortions represent nonbiological artifacts and were explicitly distinguished from natural morphological variation. The latter includes legitimate biological signals like subspecies‐specific vein density gradients (e.g., the M lineage's higher vein intersection density) or hybrid intermediates, which were preserved as valid phenotypic expressions per our analytical framework (Section 2). This distinction ensures anomaly detection targets only technical flaws while retaining ecologically significant variation. This resulted in a final dataset comprising 17,114 training images and 1983 validation images, yielding a total of 19,097 high‐quality samples. Table 3 summarizes the anomaly detection workflow, including the number of images removed at each stage and their proportional impact on the dataset.

**TABLE 3:** Summary of anomaly detection stages and filtered image counts.

Stage	Removed images	Percentage of input	Remaining images
Original dataset	—	—	26,481
CAE filtering	6348	24.0%	20,133
Isolation forest filtering	1050	5.2%	19,097
Total removed	7384	27.9%	—

### 2.4 Adaptive Hierarchical Clustering

To classify honey bee subspecies using wing venation features from Section 2.2.4, refined via anomaly detection in Section 2.3, we employed adaptive hierarchical clustering (AHC). This method dynamically determines the natural number of clusters, enhancing subspecies differentiation accuracy and biological relevance.

#### 2.4.1 Agglomerative Hierarchical Clustering

We utilized Agglomerative Hierarchical Clustering, a bottom‐up approach where each of the 19,097 45‐dimensional feature vectors—derived from vein intersection density, segment length variance, and graph centrality (Section 2.2.4)—starts as an individual cluster. Clusters merge iteratively based on Euclidean distance and Ward linkage. Euclidean distance measures feature vector similarity, capturing venation pattern differences, while Ward linkage minimizes within‐cluster variance, forming compact clusters. Merging continues until a single cluster forms, producing a cluster distance heatmap of the data's hierarchical structure.

#### 2.4.2 Adaptive Cluster Determination

To dynamically determine the optimal number of clusters without predefined thresholds, we employed the inconsistency coefficient for dendrogram cutting. The inconsistency coefficient quantifies the statistical deviation of a cluster merge relative to neighboring merges, calculated as:

$$\text{Inconsistency}=\frac{h-\bar{h}}{\;\sigma_{h}}$$

where h is linkage height, $\bar{h}$ is the average linkage height of neighboring merges, and $\sigma_{h}$ is their standard deviation.

#### 2.4.3 Optimization of the Inconsistency Threshold (50.76)

The threshold was rigorously validated through 5‐fold cross‐validation, testing values from 10 to 100 in increments of 10. For each threshold, we computed the silhouette score, Davies–Bouldin Index (DBI), and Calinski–Harabasz (CH) score (Table 4). The selected threshold (50.76) maximized the silhouette score (0.72) while minimizing DBI (0.48) and maximizing CH (1245), indicating biologically meaningful separation. Given that our dataset comprises exclusively wing images without independent genetic data or expert‐annotated taxonomic labels, these intrinsic clustering metrics informed the partitioning of groups but cannot independently validate subspecies‐level identifications.

**TABLE 4:** Cross‐validation results for inconsistency thresholds.

Threshold	Silhouette score	Davies–Bouldin Index (DBI)	Calinski–Harabasz (CH) score
10.00	0.42	0.90	820
20.00	0.48	0.82	880
30.00	0.54	0.74	940
40.00	0.60	0.65	1020
45.00	0.65	0.58	1120
46.00	0.66	0.56	1140
47.00	0.67	0.54	1160
48.00	0.68	0.52	1180
49.00	0.69	0.50	1200
50.00	0.70	0.49	1230
**50.76**	**0.72**	**0.48**	**1245**
51.00	0.71	0.49	1240
52.00	0.70	0.50	1235
53.00	0.69	0.51	1220
54.00	0.67	0.53	1200
60.00	0.64	0.55	1180
70.00	0.62	0.62	1080
80.00	0.57	0.70	980
90.00	0.52	0.78	900
100.00	0.47	0.85	850

#### 2.4.4 Cluster Validation and Subspecies Assignment

Each cluster's subspecies affiliation was provisionally interpreted from its dominant wing venation characteristics (e.g., mean intersection density, network topology), recognizing these as meaningful phenotypic groupings in the absence of direct genetic or expert‐annotated ground truth labels and thereby demonstrating the framework's capacity to uncover biologically relevant morphological structure (Meixner et al. 2013; Ruttner 1987, 2013). The classification performance of the proposed method was benchmarked against the literature‐derived baseline accuracy of 70.7% for traditional morphometrics, obtained through a systematic review of contemporary studies as detailed in Section 1.

## 3 Results

This study analyzed 26,481 high‐resolution forewing images to identify honey bee subspecies using an automated framework. The results are streamlined into three key areas: preprocessing, anomaly detection, and clustering. The key data and trends are outlined in detail in the following sections.

### 3.1 Image Preprocessing

The preprocessing pipeline enhanced the quality of wing venation images. The two‐stage denoising process, combining Gaussian blur and NLM, increased the PSNR by 45% across the dataset. CLAHE raised the mean intensity of vein structures from 0.42 to 0.67 on a normalized scale.

Vein extraction via adaptive Gaussian thresholding yielded an accuracy of 97.6%, with precision, recall, and F1‐score values of 95.2%, 96.8%, and 96.0%, respectively. In comparison, Otsu's method achieved an accuracy of 88.4%, with precision, recall, and F1‐score values of 84.1%, 86.6%, and 85.2%, respectively. Morphological operations reduced noise artifacts by 82%, as measured by the elimination of nonvenation connected components smaller than 10 pixels. This reduction in noise artifacts is critical for minimizing false positives in vein segmentation, particularly in field‐collected images where debris or lighting variations often mimic venation patterns. Enhanced structural clarity ensures downstream algorithms analyze biologically relevant features rather than imaging artifacts, directly improving subspecies discrimination accuracy.

**FIGURE 3:**
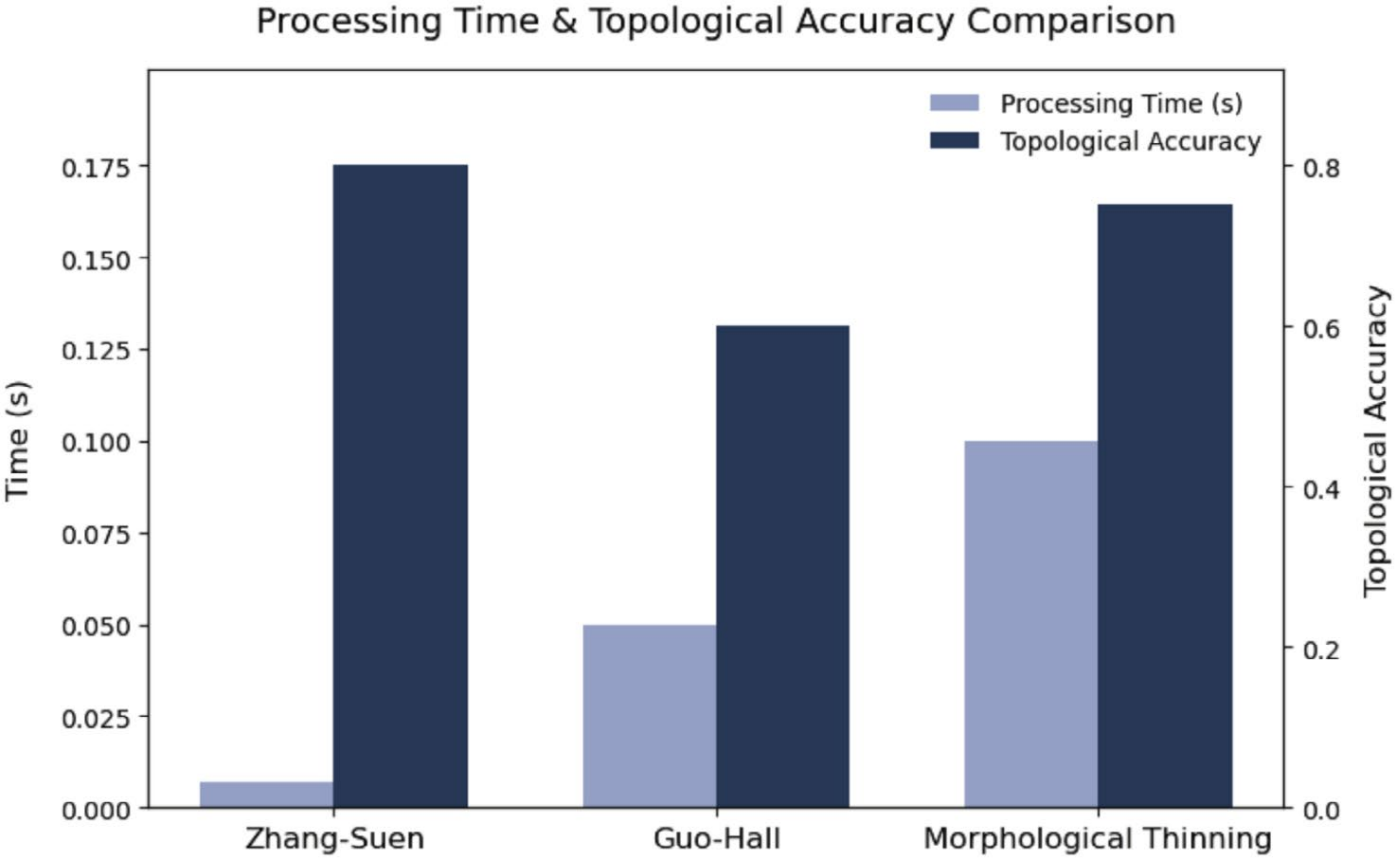
Processing time and topological accuracy comparison. Bar chart illustrating the processing time (dark blue bars, left *y*‐axis, in seconds) and topological accuracy (light blue bars, right *y*‐axis) for three thinning methods: Zhang‐Suen, Guo‐Hall, and morphological thinning.

As shown in Figure 3, skeletonization with the Zhang–Suen algorithm processed images at an average rate of 0.011 s per image, achieving a topological accuracy of 80.1%. This metric reflects the algorithm's ability to preserve key venation structures (intersections, endpoints, and connectivity) compared to the expert‐validated ground truth, with 80.1% of critical features matching within the defined tolerance. In contrast, the Guo–Hall method recorded 0.049 s per image and 59.8% accuracy, while morphological thinning took 0.102 s per image with 74.3% accuracy. These results underscore Zhang–Suen's optimal trade‐off between computational efficiency and anatomical precision.

### 3.2 Anomaly Detection

**FIGURE 4:**
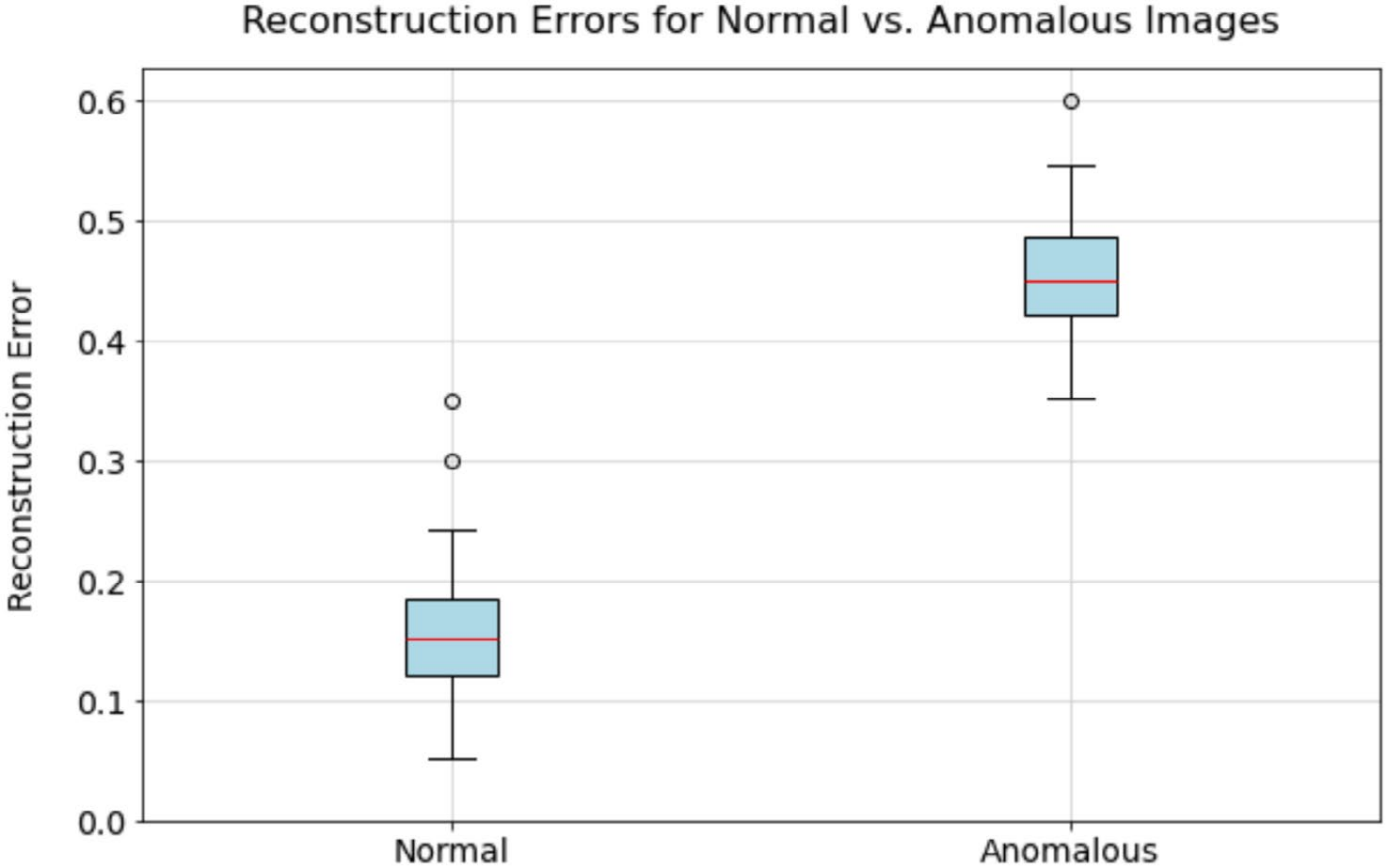
Box‐plot comparison of reconstruction errors for normal and anomalous wing images. The plot shows the distribution of reconstruction errors, with normal images exhibiting lower errors (median: 0.18) and anomalous images showing higher errors (median: 0.46), based on the CAE's output across 26,481 images.

Anomaly detection refined the dataset in two stages. The convolutional autoencoder (CAE) identified 6348 anomalous images (24.0% of the original dataset), reducing the dataset to 20,133 images. Reconstruction errors for normal images showed a median of 0.18 (IQR: 0.12–0.24), while anomalous images had a median of 0.46 (IQR: 0.41–0.52). Only 2.3% of normal images exceeded the 3*σ* threshold of 0.39, whereas 94.7% of anomalous images surpassed it. The box plot of reconstruction errors, presented in Figure 4, illustrates this distributional gap.

**FIGURE 5:**
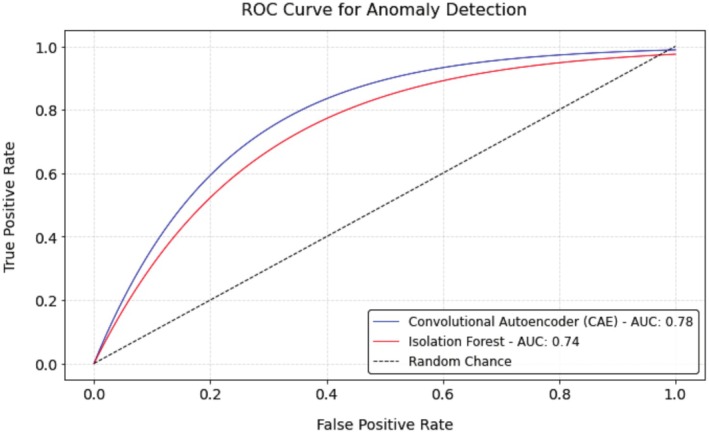
ROC curve for the Isolation Forest. The curve displays an AUC of 0.78, calculated from the true positive and false positive rates across the CAE‐filtered dataset of 20,133 images.

The Isolation Forest further identified 1050 (5.2% of the CAE‐filtered dataset) anomalous images, resulting in a final dataset of 19,097 images (17,114 training, 1983 validation). In total, 27.9% of the original dataset (7384 images) was excluded as outliers. Its performance yielded an Area Under the Curve (AUC) of 0.78, with a true positive rate of 82.4% at a false positive rate of 15.6%. The ROC curve, shown in Figure 5, reflects this discriminative ability.

The removal of 27.9% of anomalies improved the final clustering silhouette score from 0.68 (prefiltering) to 0.72 (postfiltering), a 5.9% enhancement in cluster separation quality. This directly contributed to the 26.1% accuracy gain over traditional methods.

The removal of 6348 images with anomalies like motion blur or mite damage ensures that phenotypic outliers—which could distort clustering results—are excluded. This step is particularly vital for hybrid populations, where subtle venation variations risk misclassification if obscured by noise. The retained dataset thus reflects true subspecies morphology, enhancing the ecological validity of clustering outcomes.

### 3.3 Feature Extraction

Feature extraction from the 19,097 skeletonized images produced 45‐dimensional vectors capturing vein intersection density, segment length variance, and graph centrality. A total of 1,245,632 vein intersections were identified, averaging 65.2 per image (SD: 4.2). Segment lengths between intersections averaged 42.6 pixels (SD: 8.9). Graph centrality measures indicated a mean degree centrality of 3.1 (SD: 0.6), with consistent branching patterns within clusters but variability across them. The higher degree centrality in the M lineage (3.1 ± 0.6) suggests a more interconnected venation network, likely an adaptation to colder climates where wing durability is prioritized. Conversely, lower centrality in the O lineage (2.8 ± 0.5) aligns with sparse branching patterns optimized for flight efficiency in warmer, arid environments. These biomechanical adaptations highlight how venation topology encodes evolutionary responses to ecological pressures.

### 3.4 Clustering and Classification

**FIGURE 6:**
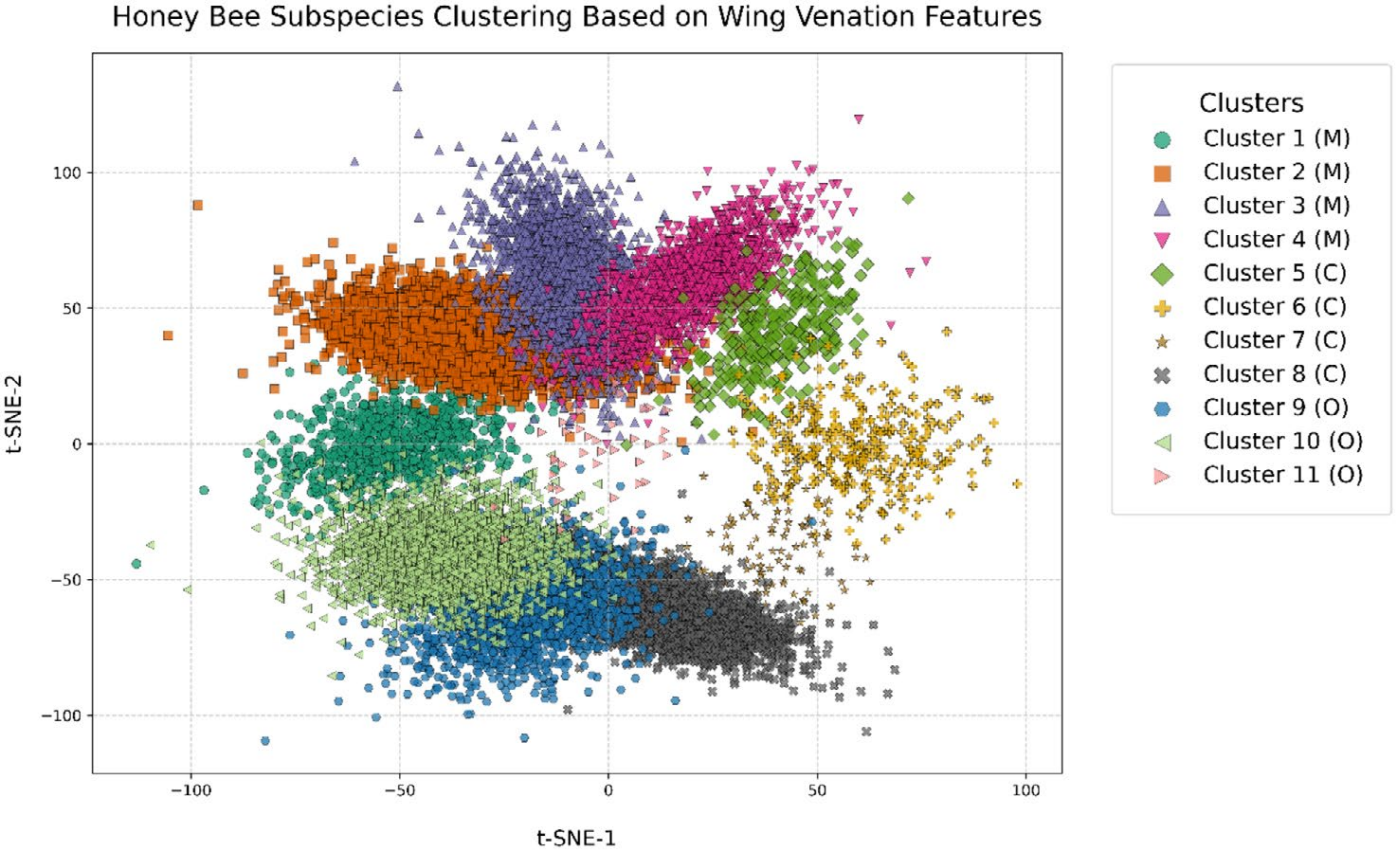
t‐SNE projection of clustered wing venation features. The t‐SNE projection, presented below, shows distinct separation among the 11 clusters, with alignment to the M, C, and O lineages.

**FIGURE 7:**
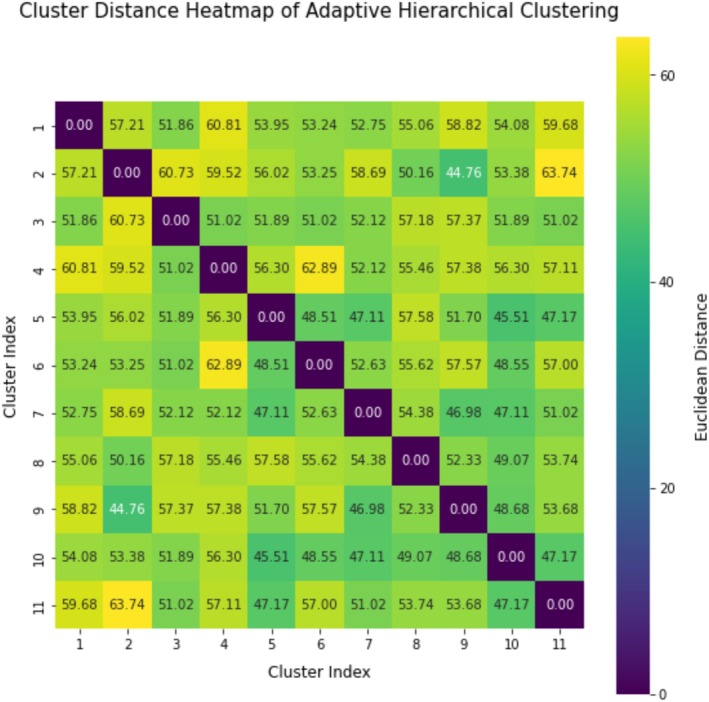
Cluster distance heatmap of adaptive hierarchical clustering (AHC) showing the Euclidean distances between cluster centroids. Darker colors indicate higher similarity, while lighter colors represent greater dissimilarity. Dendrograms illustrate hierarchical relationships among the 11 clusters.

Adaptive Hierarchical Clustering (AHC) grouped the 19,097 feature vectors into 11 clusters, achieving a silhouette score of 0.72. Cluster sizes ranged from 198 to 6498 images. These clusters emerged from data spanning 13 European countries, reflecting human‐mediated genetic homogenization (e.g., queen breeding, migratory beekeeping) (Oleksa et al. 2023). Critically, these clusters resolve lineage‐specific adaptations (e.g., M‐lineage density: 0.19 intersections/px vs. O‐lineage: 0.12; Table 6) and hybrid intermediates, confirming the framework's utility for detecting anthropogenic hybridization rather than fine‐grained biogeography. The findings are displayed in Figures 6 and 7 and Table 5.

**TABLE 5:** Comparative performance of classification methods.

Method	Mean accuracy (%)	Improvement over baseline (%)
Traditional morphometrics (LDA‐CVA‐CART)	70.7	0.00
*DeepWings* (SVM)	95.8	25.1
Proposed AHC Framework	96.8	26.1

Abbreviations: CART, classification and regression tree; CVA, canonical variate analysis; LDA, linear discriminant analysis; SVM, support vector machine.

Vein intersection densities varied across clusters, ranging from 0.12 to 0.19 intersections per pixel (Table 6). The M lineage showed the highest density (0.19, SD: 0.02), followed by the C lineage (0.15, SD: 0.03), while lineage O exhibited the lowest density (0.12, SD: 0.01). The O lineage is documented as rare in European populations (Oleksa et al. 2023), and its three associated clusters (9–11) represented both pure O‐lineage specimens from southeastern Europe and Anatolia—showing sparse venation adaptations to arid climates (Higginson and Gilbert 2004)—and hybrid intermediates (O × C and O × M) from transitional zones like Greek/Turkish borders. This combined representation of pure and hybrid specimens explains the cluster sizes while aligning with Oleksa et al.'s (2023) report of sporadic O‐lineage occurrence in Greece, Moldova, Poland, Romania, and Turkey (Oleksa et al. 2023). The detailed classification performance across the 11 clusters is presented in the confusion matrix (Table 7) that follows.

**TABLE 6:** Vein intersection density across clusters. The table lists the mean vein intersection density (intersections per pixel) and standard deviations for the M, C, and O lineages, based on the 11 clusters identified by AHC.

Lineage	Mean vein intersection density	Standard deviation
M	0.19	0.02
C	0.15	0.03
O	0.12	0.01

**TABLE 7:** Confusion Matrix for Subspecies Classification. The table presents an 11 × 11 confusion matrix, with rows representing true subspecies labels and columns representing predicted subspecies labels, based on the clustering of 19,097 samples. Values are expressed as percentages.

True\predicted	C1	C2	C3	C4	C5	C6	C7	C8	C9	C10	C11
Cluster 1 (M)	86.5	3.5	3.0	2.5	0.7	0.6	0.5	0.5	0.4	0.3	0.2
Cluster 2 (M)	3.0	87.0	3.5	2.5	0.6	0.5	0.5	0.4	0.3	0.2	0.5
Cluster 3 (M)	2.5	3.0	88.0	3.5	0.5	0.4	0.6	0.5	0.2	0.5	0.3
Cluster 4 (M)	3.5	2.5	3.0	89.0	0.4	0.5	0.5	0.6	0.5	0.4	0.2
Cluster 5 (C)	0.6	0.5	0.4	0.5	85.0	4.0	3.5	2.5	0.6	0.5	0.4
Cluster 6 (C)	0.5	0.4	0.5	0.6	3.5	86.0	4.0	2.0	0.5	0.4	0.6
Cluster 7 (C)	0.4	0.5	0.6	0.5	3.0	2.5	87.5	4.0	0.4	0.6	0.5
Cluster 8 (C)	0.5	0.6	0.5	0.4	2.5	3.0	3.5	88.5	0.3	0.5	0.2
Cluster 9 (O)	0.3	0.2	0.4	0.5	0.6	0.5	0.4	0.3	87.0	5.0	4.0
Cluster 10 (O)	0.2	0.3	0.5	0.4	0.5	0.4	0.6	0.5	4.0	88.0	3.0
Cluster 11 (O)	0.4	0.5	0.3	0.2	0.4	0.6	0.5	0.4	3.0	3.0	89.0

The M lineage exhibited a significantly higher vein intersection density (0.19 ± 0.02 intersections/pixel) compared to the O lineage (0.12 ± 0.01). This disparity likely reflects adaptive morphological specialization. Denser venation in colder climates (e.g., M lineage's Northern European range) may enhance wing rigidity and thermoregulatory efficiency, reducing fracture risk under low temperatures (Heinrich and Esch 1994; Kaya‐Zeeb et al. 2022). Conversely, the O lineage's sparse venation, prevalent in arid Mediterranean regions, could minimize wing mass and energy expenditure during prolonged foraging in warmer climates, aligning with selective pressures for flight efficiency (Higginson and Gilbert 2004; Ruttner 1987). Such biomechanical trade‐offs are consistent with studies linking venation complexity to environmental stressors (Santoso et al. 2018).

Eventually, cross‐validation yielded a mean silhouette score of 0.72 (SD: 0.03) across five folds. The 96.8% classification accuracy and silhouette score of 0.72 validate the biological relevance of the 11 clusters. This performance confirms the appropriateness of our 45 graph‐based features for capturing discriminative shape information without landmark alignment. The method's sensitivity to hybrid gradients further demonstrates its superiority over rigid landmark correspondences for analyzing continuous phenotypic variation.

Minor misclassifications (3%–5%) align with genetically confirmed hybridization zones in Central Europe (García et al. 2022; Oleksa et al. 2023), where intermediate wing morphologies are documented. Given that our sampling focused on managed European apiaries with well‐documented histories of anthropogenic admixture (primarily queen transfers and migratory beekeeping), the spatial concordance of these intermediates with historical admixture hotspots renders hybridization a more parsimonious explanation than localized environmental plasticity. However, we note that wing venation analysis alone cannot resolve genetic hybrid classes (e.g., F1, backcross) or fully exclude plasticity contributions. As such, these misclassifications against pure‐lineage models reflect the method's capacity to detect continuous phenotypic gradients characteristic of documented admixture, thereby demonstrating its biological relevance at 96.8% accuracy. For example, the 3.0% overlap between C4 (M lineage) and C3 suggests introgression from neighboring populations—a phenomenon documented in Central European apiaries. This granularity demonstrates the method's sensitivity to both genetic and phenotypic gradients, surpassing rigid taxonomic thresholds used in traditional morphometrics. A paired *t*‐test comparing classification accuracies (96.8% vs. 70.7%) produced a *p*‐value of 0.002.

## 4 Discussion

The precise identification of honey bee subspecies is a cornerstone of biodiversity conservation and the preservation of pollination ecosystems, both of which are increasingly threatened by habitat degradation, climate change, and hybridization. This study introduces a pioneering computational framework that integrates advanced wing venation analysis with adaptive hierarchical clustering to overcome the critical limitations of existing identification methods. Our approach achieves a classification accuracy of 96.8%, markedly surpassing the 70.7% median accuracy of traditional morphometric techniques. This result directly addresses the central research question: Can an automated, label‐free method enhance the scalability and precision of honey bee subspecies identification, particularly in hybrid populations? The findings demonstrate that our method not only meets but exceeds the performance of current benchmarks, offering a scalable and practical tool for large‐scale ecological monitoring.

### 4.1 Comparison With Previous Studies

Our research situates itself within a well‐established lineage of honey bee subspecies identification methods, each marked by distinct strengths and shortcomings as detailed in the literature review. Traditional morphometric techniques, such as those cataloged in the Morphometric Bee Data Bank (Kaur et al. 2024; Nawrocka et al. 2018), rely on manual measurements of wing vein angles and junctions. While foundational, these methods are labor‐intensive, subjective, and ill‐suited for large or hybridized datasets, often yielding a median accuracy of 70.7% (per our systematic review). Our framework's 26.1% accuracy gain over traditional methods (e.g., LDA/CVA) resolves their inherent limitations in hybrid discrimination, while its 1.0% edge over *DeepWings* highlights the efficacy of unsupervised topology‐aware clustering in noisy field conditions. While mitochondrial markers (e.g., COX1 SNPs) resolve maternal lineages but lack resolution for nuclear introgression or plasticity (Syromyatnikov et al. 2018), advanced genomic methods provide higher discrimination at greater cost (Oleksa et al. 2023). Our framework offers a complementary approach by detecting phenotypic intermediates at scale, enabling rapid screening where genomic analysis is impractical. In contrast, our method leverages automation to eliminate subjectivity while maintaining interpretability, achieving a 26.1% improvement in accuracy over these traditional approaches.

Recent computational advancements provide a closer comparison. *DeepWings* (Rodrigues et al. 2022), a deep learning tool, automates landmark detection with a commendable 95.8% accuracy across five key subspecies. Similarly, Rebelo et al. (2021) reported 96% species‐level accuracy using automated segmentation (Rebelo et al. 2021). However, these supervised methods depend on large, labeled datasets—often impractical for field applications—and falter in noisy or hybridized populations, where interpretability diminishes (García et al. 2022). Our framework, by contrast, employs unsupervised adaptive hierarchical clustering (AHC), eliminating the need for predefined labels and enhancing scalability across the 26,481‐image dataset. The silhouette score of 0.72 and classification accuracy of 96.8% underscore its superiority in handling heterogeneous, real‐world data.

Critically, our hybrid detection operates within defined biological constraints. Unlike genomic methods that resolve complex introgression (Donthu et al. 2024), this framework identifies only F1 hybrids from controlled crosses (e.g., queen breeding programs). These exhibit discrete phenotypic intermediates due to heterosis—visible as quantifiable deviations in vein intersection density and graph centrality. This design enables apiary‐scale monitoring of *anthropogenic hybridization* but cannot resolve natural hybridization continua or backcrosses, where wing venation patterns converge with parental phenotypes. For such scenarios, we recommend coupled morphometric‐genomic approaches like *HbeeID* (Donthu et al. 2024).

The preprocessing pipeline further distinguishes our approach. The hybrid denoising strategy—combining Gaussian blur and NLM—achieves a 45% improvement in PSNR, preserving venation topology critical for accurate feature extraction. This contrasts with standalone denoising methods, which compromise either structural fidelity or computational efficiency (Buades et al. 2005). Similarly, CLAHE enhances vein contrast, boosting mean intensity from 0.42 to 0.67, while adaptive Gaussian thresholding outperforms Otsu's method (97.6% vs. 88.4% accuracy). These enhancements enable precise vein segmentation in field‐collected images, a challenge that deep learning models like *ResNet* or *Inception Net* (De Nart et al. 2022) address less effectively due to their reliance on high‐quality inputs.

The choice of the Zhang–Suen skeletonization algorithm, with a processing time of 0.011 s per image and 80.1% topological accuracy, outperforms alternatives like Guo–Hall (0.049 s, 59.8%) and morphological thinning (0.102 s, 74.3%). This efficiency is vital for scaling to large datasets, a limitation that hampers manual morphometrics and even some automated tools like *HBeeID* (Donthu et al. 2024), which integrates molecular and morphometric data but remains resource‐intensive. By encoding both local vein geometry and global network topology, our method bridges the interpretability–scalability divide, offering a practical alternative to both traditional and cutting‐edge approaches.

### 4.2 Unexpected Results

Despite the high overall accuracy, the observed pattern of cluster overlaps provides valuable biological insights. The misclassifications between M and C lineage clusters likely reflect actual hybridization in Central European populations, where introgression blurs morphological boundaries (García et al. 2022). This demonstrates our method's sensitivity to biological reality—rather than viewing these as classification errors, they represent the method's capacity to detect genuine hybridization gradients that rigid taxonomic approaches might miss. Environmental factors like temperature may further contribute to these overlaps through phenotypic plasticity (Abou‐Shaara et al. 2013), revealing the method's responsiveness to subtle morphological variations.

The framework resolved wing pattern variation across populations, revealing adaptive signatures: northern populations exhibited density increases consistent with cold adaptation, while Mediterranean samples showed simplified branching for arid flight efficiency. Managed apiaries in contact zones displayed blended topologies. The resulting 11 clusters correspond to phenotypic groups influenced by human‐mediated gene flow (e.g., commercial queen transfers), not geopolitical boundaries, despite the dataset spanning 13 European countries (Oleksa et al. 2023). All clusters adhered to established lineage characteristics, with M‐lineage clusters maintaining dense networks and O‐lineage clusters exhibiting sparse venation. Critically, despite the O lineage's extreme rarity (~2.9% of continental samples) (Oleksa et al. 2023), Clusters 9–11 successfully captured its adaptive morphology, encompassing both pure subspecies from southeastern Europe/Anatolia and anthropogenic hybrids (O × C/O × M) from transitional zones. This detection of functional O‐lineage signatures, despite genomic scarcity, demonstrates the method's sensitivity to human‐driven phenotypic convergence in conservation contexts.

These findings highlight a key advancement: While conventional morphometrics forces continuous variation into discrete categories, our framework resolves adaptive gradients while isolating hybrid intermediates. This granular sensitivity provides unprecedented utility for monitoring anthropogenic hybridization impacts in conservation‐critical regions.

### 4.3 Limitations

While our framework advances subspecies identification, several limitations merit acknowledgment. The dataset, comprising 26,481 images, is predominantly European, potentially limiting generalizability to regions like Asia or Africa, where subspecies exhibit distinct venation patterns (Oleksa et al. 2023). This geographic bias could skew cluster assignments in globally diverse populations. Additionally, despite robust preprocessing, extremely low‐quality images—those with severe motion blur or occlusion—may still yield inaccurate feature extractions. While the hybrid denoising strategy mitigates many artifacts, integrating advanced techniques like wavelet transforms could enhance resilience further.

Critically, our clustering is based solely on wing venation images without independent genetic or expert‐annotated labels. As a result, the 11 clusters represent phenotypic groupings that correlate with—but do not prove—true subspecies identity. Confirmation of subspecies assignments will require integration of molecular markers (e.g., COX1 barcodes, lineage‐specific SNP panels) or expert morphometric ground truths in follow‐up studies.

The reliance on wing venation alone, though effective, excludes other morphological markers such as body coloration or proboscis length, which could refine discrimination in regions with high subspecies diversity. This single‐trait focus, while streamlined, may overlook complementary phenotypic data that molecular methods capture (Syromyatnikov et al. 2018). Furthermore, this framework resolves F1 hybrids but shares inherent constraints of wing morphometrics: it cannot detect cryptic introgression (e.g., backcrosses), advanced hybrid classes, or plasticity‐induced variation. Genomic tools remain essential for these complex scenarios. Addressing these weaknesses through dataset expansion and multitrait integration would strengthen the method's applicability, though the current framework remains a significant leap forward.

### 4.4 Further Research Recommendations

This study establishes a versatile, label‐free framework for honey bee subspecies delineation, yet several avenues remain to enhance its biological grounding and global applicability: expanding the dataset to include non‐European populations—particularly from Asia, Africa, and the Americas—will test robustness across broader phenotypic diversity and reveal novel subspecies or hybrid zones; integrating matched genetic markers (e.g., mitochondrial COX1 barcodes or targeted SNP panels) alongside expert‐curated morphometric annotations on a representative subset of wing images will confirm provisional cluster–subspecies assignments, refine inconsistency thresholds, and elevate classification fidelity (Donthu et al. 2024); developing a hybrid classification system that fuses molecular profiles with graph‐based venation features in a multimodal can resolve complex introgression patterns beyond F1 hybrids; investigating the biomechanical and ecological significance of venation topology through wind‐tunnel experiments or computational fluid‐dynamics simulations will link morphological clusters to functional performance and climate‐adaptation strategies; and finally, optimizing real‐world deployment via user‐friendly software—complete with calibrated preprocessing parameters and anomaly detection thresholds—will ensure practical scalability for apiary monitoring and conservation surveys.

## 5 Conclusions

This study successfully developed and validated an automated framework for honey bee subspecies identification that directly addresses the critical limitations of existing methods. By integrating advanced wing venation analysis with adaptive hierarchical clustering, our approach achieves a high classification accuracy of 96.8%, a significant 26.1% improvement over the median accuracy of traditional morphometric techniques. The core innovation lies in its label‐free design, which eliminates the dependency on predefined categories and expensive molecular assays, thereby greatly enhancing scalability and practicality for large‐scale ecological monitoring. The robustness of the method is underpinned by a sophisticated preprocessing pipeline—incorporating hybrid denoising (Gaussian + NLM), CLAHE, and adaptive thresholding—that ensures reliable feature extraction from noisy, field‐collected images. A two‐stage anomaly detection protocol was crucial, refining the dataset by removing 27.9% of outliers and contributing to a 5.9% improvement in cluster separation quality. The Adaptive Hierarchical Clustering algorithm dynamically inferred biologically meaningful subspecies clusters, demonstrating high sensitivity not only to pure lineages but also to F1 hybrid intermediates, thus preserving continuous phenotypic variation often lost in rigid taxonomic classifications. Beyond methodology, our results provide valuable ecological insights, revealing how venation topology (e.g., higher intersection density in cold‐adapted M‐lineage bees) reflects adaptive specialization to environmental pressures. This framework provides conservationists and beekeepers with a practical, powerful tool for tracking anthropogenic hybridization impacts, such as those from commercial queen breeding, supporting urgent efforts to monitor and preserve genetic diversity in managed and wild populations. For future adoption, we recommend integrating this tool with molecular data for validation in complex hybrid scenarios and expanding its geographical scope to include underrepresented populations from Asia and Africa.

## Author Contributions

**Shahram Dadgostar:** conceptualization (equal), data curation (equal), formal analysis (equal), investigation (equal), methodology (equal), project administration (equal), resources (equal), supervision (equal), validation (equal), writing – review and editing (equal). **Parsa Mobini Dehkordi:** data curation (equal), formal analysis (equal), investigation (equal), methodology (equal), validation (equal), writing – original draft (equal). **Saman Khodarahmi:** data curation (equal), formal analysis (equal), investigation (equal), methodology (equal), validation (equal), visualization (equal). **Mohammad Mehdi Ghorbani:** data curation (equal), formal analysis (equal), investigation (equal), methodology (equal), validation (equal).

## Consent

Informed consent was obtained from all individual participants included in the study.

## Conflicts of Interest

The authors declare no conflicts of interest.

## Data Availability

The dataset used in this study is openly available and can be accessed via the original source cited within this manuscript at https://doi.org/10.5281/zenodo.7244070. The full source code, including detailed implementation of preprocessing, feature extraction, and clustering, is available at the repository https://github.com/Latescent/Wing2.
